# Dietary Chrysin Suppresses Formation of Actin Cytoskeleton and Focal Adhesion in AGE-Exposed Mesangial Cells and Diabetic Kidney: Role of Autophagy

**DOI:** 10.3390/nu11010127

**Published:** 2019-01-09

**Authors:** Eun-Jung Lee, Min-Kyung Kang, Yun-Ho Kim, Dong Yeon Kim, Hyeongjoo Oh, Soo-Il Kim, Su Yeon Oh, Young-Hee Kang

**Affiliations:** Department of Food and Nutrition, Hallym University, Chuncheon, Kangwon-do 24252, Korea; reydmswjd@naver.com (E.-J.L.); mitholy@hallym.ac.kr (M.-K.K.); royalskim@hallym.ac.kr (Y.-H.K.); ehddus3290@naver.com (D.Y.K.); ohhyeongju@gmail.com (H.O.); ky4850@naver.com (S.-I.K.); suy0411@naver.com (S.Y.O.)

**Keywords:** actin cytoskeleton, advanced glycation end products, autophagy, chrysin, focal adhesion, mesangial migration

## Abstract

Advanced glycation end products (AGE) play a causative role in the development of aberrant phenotypes of intraglomerular mesangial cells, contributing to acute/chronic glomerulonephritis. The aim of this study was to explore mechanistic effects of the flavonoid chrysin present in bee propolis and herbs on actin dynamics, focal adhesion, and the migration of AGE-exposed mesangial cells. The in vitro study cultured human mesangial cells exposed to 33 mM glucose and 100 μg/mL AGE-bovine serum albumin (AGE-BSA) for up to 5 days in the absence and presence of 1–20 μM chrysin. The in vivo study employed *db/db* mice orally administrated for 10 weeks with 10 mg/kg chrysin. The presence of ≥10 μM chrysin attenuated mesangial F-actin induction and bundle formation enhanced by AGE. Chrysin reduced the mesangial induction of α-smooth muscle actin (α-SMA) by glucose, and diminished the tissue α-SMA level in diabetic kidneys, indicating its blockade of mesangial proliferation. The treatment of chrysin inhibited the activation of vinculin and paxillin and the induction of cortactin, ARP2/3, fascin-1, and Ena/VASP-like protein in AGE-exposed mesangial cells. Oral administration of chrysin diminished tissue levels of cortactin and fascin-1 elevated in diabetic mouse kidneys. Mesangial cell motility was enhanced by AGE, which was markedly attenuated by adding chrysin to cells. On the other hand, chrysin dampened the induction of autophagy-related genes of beclin-1, LC3 I/II, Atg3, and Atg7 in mesangial cells exposed to AGE and in diabetic kidneys. Furthermore, chrysin reduced the mTOR activation in AGE-exposed mesangial cells and diabetic kidneys. The induction of mesangial F-actin, cortactin, and fascin-1 by AGE was deterred by the inhibition of autophagy and mTOR. Thus, chrysin may encumber diabetes-associated formation of actin bundling and focal adhesion and mesangial cell motility through disturbing autophagy and mTOR pathway.

## 1. Introduction

Mesangial cells possess irregular structures comprised of flattened cylinder-like cell bodies and contain actin, myosin, and α-actinin at both ends, granting them contractile features [1]. The anchoring filaments from mesangial cells to the glomerular basement membrane can influence glomerular capillary blood flow [2,3]. The structural support of mesangial cells in kidney glomeruli counteracts the expansible forces created by pressure gradients in capillary vessels and manipulates the capillary blood flow [4]. Glomerular mesangial cells migrate in response to platelet-derived growth factor (PDGF) and angiotensin II, which is crucial for glomerulopathy and glomerular development [5,6]. There is emerging evidence for the role of the abnormal migratory polarity of mesangial cells due to glomerular injury in the pathogenesis of proliferative glomerulonephritis [4,7,8]. Glomerular injury-triggered molecular changes in mesangial cells in glomerulonephritis still remain elusive. In general, directional cell migration entails the establishment of cytoskeletal alterations that are essential for actin polarization and focal adhesion turnover [9,10]. In the same context, the migratory process is controlled by coordinated actin dynamics and focal adhesion turnover at the peripheral ruffles in migrating mesangial cells [6]. However, identifying the key molecular players in motility and clear-cut molecular functions remains challenging.

Glucose toxicity is a primary cause of glomerular injury in diabetic kidney diseases, and involves the production of advanced glycation end products (AGE) [11]. Increased glucose levels result in glomerular oxidative stress via activation of metabolic pathways, which consequently produces and accumulates AGE, leading to glomerular injury [12,13]. The activation of the AGE-receptor for advanced glycation end products (RAGE) influences endothelial actin cytoskeletal rearrangement and dysfunction [14,15]. A recent report shows that AGE-induced oxidative stress stimulates proliferation and migration of vascular smooth muscle cells [16]. Dynamics of focal adhesions containing vinculin and paxillin is entailed for cell polarization and motility, and extracellular matrix remodeling [17,18]. Cell migration entails coordination between focal adhesion and actin cytoskeleton via F-actin-binding vinculin abundant in integrin-based focal adhesions [17]. Exposure of retinal pericytes to AGE induces cell migration via phosphorylation of focal adhesion kinase and paxillin [19]. Connective tissue growth factor prompts mesangial cell migration via disassembly of focal adhesion complexes and activation of cell polarization [20]. On the other hand, autophagy is responsible for the degradation of AGE by the upregulation of lysosomal biogenesis and function in diabetic nephropathy (DN) [21]. Accordingly, strategies aimed at enhancing the lysosomal function of autophagy-related proteins can hold promise for treating DN. One study shows that autophagy is likely to be a mechanism triggered to repair the reactive oxygen species-induced loss of the AGE-treated cells and thereby prompts cell survival [22]. 

**Figure 1:**
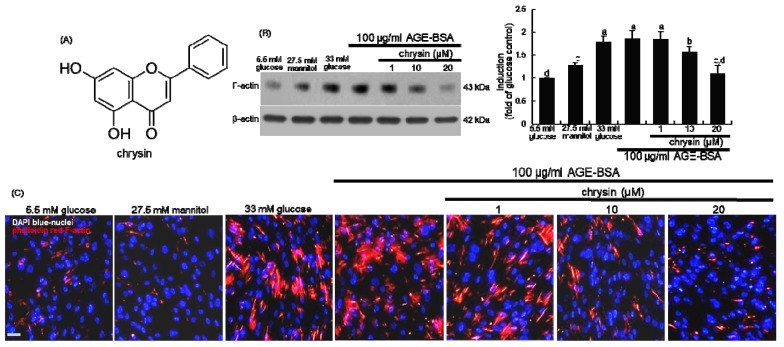
Chemical structure of chrysin (**A**), and inhibition of F-actin induction by chrysin in advanced glycation end products (AGE)-bovine serum albumin (BSA)-exposed human renal mesangial cells (HRMC, **B** and **C**). HRMC were challenged with 5.5 mM glucose, 5.5 mM glucose plus 27.5 mM mannitol as osmotic controls or with 33 mM glucose or 100 μg/mL AGE-BSA in the absence and presence of 1–20 μM chrysin. The F-actin induction was measured by Western blot analysis using cell lysates with a primary antibody of F-actin (**B**). β-Actin protein was used as an internal control. Representative blots shown are typical of three independent experiments. The bar graphs (mean ± SEM) in the right panel represent quantitative results obtained from a densitometer. Values not sharing a letter are different at *p* < 0.05. Red-rhodamine phalloidin staining for F-actin formation was conducted in AGE-BSA-exposed HRMC (**C**). Nuclear counter-staining was done by using blue 4’,6-diamidino-2-phenylindole. Scale bar = 50 µm. Each photograph is representative of at least four animals. Magnification: 200-fold.

Numerous studies have demonstrated that natural compounds display renoprotective effects by diverse mechanisms [23,24,25]. Polyphenolic flavonoids are known to attenuate hyperglycemia-induced renal endothelial barrier dysfunction, urinary albumin excretion, and glomerular hyperfiltration [23]. Our previous studies showed that several natural compounds counteracted renal mesangial fibrosis and inflammation, renal tubulointerstitial fibrosis, glomerulosclerosis, and podocyte injury [26,27,28]. Chrysin (5,7-dihydroxyflavone, Figure 1A), a flavonoid abundant in edible plants such as passion flowers, mushrooms, honey, and bee propolis, is known to exhibit multiple biological effects, including anti-inflammatory, anti-atherogenic, and neuroprotective properties [29,30,31]. In addition, growing evidence suggests that chrysin may display nephroprotective activities in rodents [32,33]. Thus, the present study attempted to explore the renoprotective effects of chrysin on the formation of F-actin cytoskeleton and focal adhesion complex in AGE-exposed mesangial cells and diabetic mouse kidneys. Our recent study demonstrates that chrysin may inhibit glucose-mediated AGE-associated glomerulosclerosis and fibrosis [34]. However, little is known about the renoprotective role of chrysin in actin cytoskeleton rearrangement, focal adhesion formation, and cell migration due to AGE. One investigation shows that the flavonoid myricetin suppresses retinal pericytes migration through suppressing activation of ERK1/2-focal adhesion kinase-paxillin by AGE [19]. Whether the presence of chrysin modulated mesangial cell proliferation and motility by AGE was also examined. This study further elucidated how chrysin manipulated autophagy as a molecular player involved in the actin cytoskeleton, focal adhesion assembly, and motility.

## 2. Materials and Methods

### 2.1. Chemicals

Fetal bovine serum (FBS), penicillin–streptomycin and trypsin–EDTA, were provided by BioWhittaker (San Diego, CA, USA). 3-(4, 5-Dimetylthiazol-yl)-diphenyl tetrazolium bromide (MTT) was obtained from DUCHEFA Biochemie (Haarlem, The Netherlands). Dulbecco’s modified Eagle media (DMEM), nutrient mixture F-12 Ham medium, mannitol, and D-glucose, were supplied by Sigma-Aldrich Chemical (St. Louis, MO, USA), as were all other reagents unless specifically stated otherwise. Antibodies of F-actin, α-smooth muscle actin (α-SMA), Arp2/3 antibody, mTOR, and phospho-mTOR were supplied by Abcam (Cambridge, UK). Antibodies of beclin-1, cortactin, Fascin1, and Ena/VASP (EVL) were provided by Santa Cruz Biotechnology (Dallas, TX, USA). Phospho-vinculin antibody was obtained from Biorbyt (Cambridge, UK). Atg7 antibody was purchased from Aviva system Biology (San Diego, CA, USA). Antibodies of Atg3 and phospho-paxillin were obtained from Cell Signaling Technology (Beverly, CA, USA). LC3 antibody was supplied by MBL International Corporation (Woburn, MA, USA). AGE-bovine serum albumin (AGE-BSA) was provided by Merck Millipore (Billerica, MA, USA). Horseradish peroxidase (HRP)-conjugated goat anti-rabbit IgG, goat anti-mouse, and donkey anti-goat IgG were obtained from Jackson ImmunoResearch Laboratories (West Grove, PA, USA). SB03580 (MAP kinase inhibitor) was provided from Calbiochem (Billerica, MA, USA)

Chrysin (Sigma-Aldrich Chemical, St. Louis, MO, USA) was dissolved in dimethyl sulfoxide (DMSO) for live culture with cells; a final culture concentration of DMSO was <0.5%.

### 2.2. Culture of Human Renal Mesangial Cells (HRMC) 

HRMC (Sciencell Research Laboratories, Carlsbad, CA, USA) were cultured at 37 °C humidified atmosphere of 5% CO2 in air. Routine culture of HRMC was performed in DMEM/F12 (7:1) media containing 15% FBS, 2 mM glutamine, 100 U/mL penicillin, and 100 μg/mL streptomycin. HRMC in 6-10th passage were sub-cultured at 80% confluence and used for further experiments. To mimic diabetic glomerular injury caused by chronic hyperglycemia, HRMC was incubated in 33 mM glucose- or 100 μg/mL AGE-BSA-supplemented DMEM containing 2% FBS and 2–8 μg/mL insulin for 3 days in the absence and presence of 1–20 μM chrysin. For osmotic control incubation, another set of HRMC was cultured in DMEM (5.5 mM) containing 2% FBS (+2 μg/mL insulin) and supplemented with 27.5 mM mannitol. Culture media was collected and stored at −20 °C. 

After the 3-day incubation in 33 mM glucose and 100 μg/mL AGE-BSA, the MTT assay was routinely carried out for measuring cell proliferation. After the unconverted MTT was removed, cells were dissolved in isopropanol with gentle shaking. The absorbance of formazan dye was measured at *λ* = 570 nm with background subtraction using *λ* = 690 nm. There was no cytotoxicity of 1–20 μM chrysin per se observed [34]. 

### 2.3. In Vivo Animal Experiments

Adult male *db/db* mice (C57BLKS/+Lepr^db^ Iar; Jackson Laboratory, Sacramento, CA, USA) and their age-matched non-diabetic *db/m* littermates (C57BLKS/J; Jackson Laboratory, Sacramento, CA, USA) were used in the present study. Mice were kept on a 12 h light/12 h dark cycle at 23 ± 1 °C with 50 ± 10% relative humidity under specific pathogen-free conditions, fed a standard laboratory chow diet (CJ Feed, Seoul, Korea), and were provided with water ad libitum at the animal facility of Hallym University. This study included *db/db* mice at 7 weeks of age because they begin to develop diabetes (hyperglycemia) at the age of 7–8 weeks. The animals were allowed to acclimatize for a week before beginning the experiments. Mice were divided into three subgroups (*n* = 7–9 for each subgroup). The first group of mice was non-diabetic *db/m* control mice and *db/db* mice were divided into two groups. One group of *db/db* mice was daily supplemented 10 mg/kg BW chrysin via gavage for 10 weeks. Food intake, body weight, and drinking water intake of *db/db* mice increased, relative to those of *db/m* controls. However, the supplementation of chrysin led to a decline in water drinking after the 6–7th week. The measurement of fasting blood levels of glucose and glycated hemoglobin HbA1C were conducted every other week from mouse tail veins. Chrysin treatment diminished plasma levels of glucose and HbA1C elevated in *db/db* mice. The 24 h urine samples were collected in metabolic cages during the 10-week chrysin supplementation. The urine volume was reduced in chrysin-treated mice by ~50–60%, and diabetic proteinuria was alleviated. 

All experiments were approved by the Committee on Animal Experimentation of Hallym University and performed in compliance with the University’s Guidelines for the Care and Use of Laboratory Animals (hallymR1 2016-10). No mice died and no apparent signs of exhaustion were observed during the experimental period. 

### 2.4. Western Blot Analysis

Western blot analysis was conducted using whole-cell lysates and tissue extracts prepared from HRMC at a density of 3.0 × 10^5^ cells. Whole-cell lysates and mouse renal tissue extracts were prepared in a lysis buffer containing 1 M β-glycerophosphate, 1% β-mercaptoethanol, 0.5 M NaF, 0.1 M Na_3_VO_4_, and protease inhibitor cocktail. Cell lysates and tissue extracts containing equal amounts of total proteins were electrophoresed on 6–15% SDS-PAGE and transferred onto a nitrocellulose membrane. Non-specific binding was blocked by soaking the membrane in a TBS-T buffer (50 mM Tris–HCl (pH 7.5), 150 mM NaCl, and 0.1% Tween 20) supplemented 3% BSA for 3 h. The membrane was incubated with an antibody to F-actin, α-SMA, phospho-paxillin, phospho-vinculin, cortactin, Arp2/3, fascin-1, EVL, beclin-1, LC3, Atg3, Atg7, mTOR, or phospho-mTOR. The membrane was then incubated with a secondary antibody of goat anti-rabbit IgG, goat anti-mouse IgG, or donkey anti-goat IgG conjugated to HRP. Each protein level was determined by using Supersignal West Pico Chemiluminescence detection reagents (Pierce Biotechnology, Rockford, IL, USA) and Konica X-ray film (Konica Co., Tokyo, Japan). Incubation with mouse anti-human β-actin was conducted for the comparative control. 

### 2.5. Rhodamine-Phalloidin Staining of F-Actin

HRMC (7 × 10^4^ cells) grown on 24 well glass chamber slides were exposed to 33 mM glucose or 100 μg/mL AGE-BSA in the absence and presence of 1–20 μM chrysin. Cells were fixed in 4% formaldehyde for 10 min and washed with pre-warmed phosphate-buffered saline (PBS). Subsequently, 10 units of the fluorescent dye rhodamine phalloidin were added to cells and incubated for 20 min. Nuclear staining was also conducted by using 4 mg/mL 4’,6-diamidino-2-phenylindole (DAPI). Each slide was mounted in VectaMount mounting medium (Vector Laboratories, Burlingame, CA, USA). Fluorescent images were taken with an Axiomager Optical fluorescence microscope (Zeiss, Oberkochen, Germany).

### 2.6. Mesangial Cell Motility

To assess the effects of glucose and AGE on mesangial cell motility in the absence and presence of chrysin, an in vitro scratch wound assay was employed. Briefly, mesangial cells were seeded onto a 12-well plate and incubated for 24 h in 10% FBS-containing media. Subsequently, confluent cells were scratched away horizontally on each well using a pipette tip. After scratching, injured cells were incubated for another 24 h in serum-free culture media containing 100 μg/mL AGE-BSA with and without 1–20 μM chrysin. Images of the scratches were photographed in the 2–3 microscopic fields per well using a microscope with CCD camera (Motic^®^, Wetzlar, Germany). A reduction of the scratched area indicates a sign of mesangial cell migration.

### 2.7. Immunocytochemical Staining

Immunofluorescent cytochemical staining was conducted to reveal the co-localization of Atg7 to F-actin fibers in mesangial cells grown on 24-well chamber slides. Cells were fixed with 4% formaldehyde for 20 min and permeated with 0.1% Triton X-100 for 10 min on ice. Cells were specifically blocked with 20% FBS for 1 h, and a primary antibody of Atg7 and a secondary antibody of FITC-conjugated IgG were applied to cells. Subsequently, the fluorescent dye rhodamine phalloidin was added to cells, and incubated for 20 min for F-actin staining. Nuclear counterstaining was performed with DAPI. Each slide was mounted in a mounting medium and images of each slide were taken using an optical Axiomager microscope (Zeiss, Oberkochen, Germany).

### 2.8. Data Analysis

The results are presented as mean ± SEM for each treatment group. Statistical analyses were performed using Statistical Analysis Systems statistical software package version 6.12 (SAS Institute Inc., Cary, NC, USA). Significance was determined by one-way ANOVA, followed by Duncan range test for multiple comparisons. Differences were considered significant at *p* < 0.05.

## 3. Results

### 3.1. Effect of Chrysin on the Induction of Cytoskeletal Actin Proteins

This study attempted to investigate if chrysin retarded diabetes-induced mesangial actin polymerization in kidneys. When 33 mM glucose or 100 μg/mL AGE-BSA was supplemented to mesangial cells in culture, the F-actin induction was elevated (Figure 1B). However, the elevated F-actin induction was attenuated in the presence of ≥10 μM chrysin. In addition, high glucose and AGE enhanced the F-actin formation, as evidenced by visualization with rhodamine phalloidin (Figure 1C). In contrast, the reddish staining of AGE-exposed mesangial cells was diminished by the presence of chrysin. 

**Figure 2:**
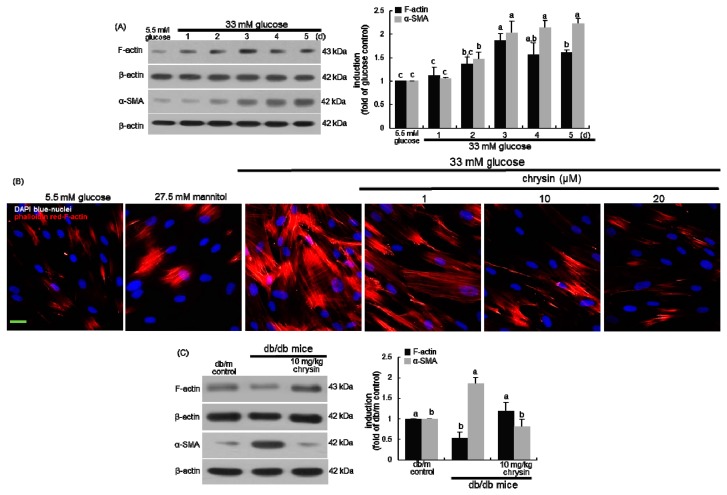
Temporal responses of protein induction of F-actin and α-SMA to glucose (**A**), inhibition of F-actin formation by chrysin in glucose-exposed human renal mesangial cells (HRMC, **B**) and tissue levels of F-actin and α-SMA in chrysin-treated diabetic kidneys (**C**). HRMC were challenged with 5.5 mM glucose, 5.5 mM glucose plus 27.5 mM mannitol as osmotic controls or with 33 mM glucose in the absence and presence of 1–20 μM chrysin. Diabetic mice were orally administrated with 10 mg/kg chrysin for 10 weeks. The induction of F-actin and α-SMA was measured by Western blot analysis using cell lysates and renal tissue extracts with a primary antibody of F-actin or α-SMA (**A**,**C**). β-Actin protein was used as an internal control. Representative blots shown are typical of three independent experiments. The bar graphs (mean ± SEM) in the right panel represent quantitative results obtained from a densitometer. Values not sharing a letter are different at *p* < 0.05. Red-rhodamine phalloidin staining for F-actin formation was conducted in high glucose-exposed HRMC (**B**). Nuclear counter-staining was done by using blue 4’,6-diamidino-2-phenylindole. Scale bar = 50 µm. Each photograph is representative of at least four animals. Magnification: 400-fold.

The temporal induction of the filamentous F-actin and the contractile α-SMA was examined in mesangial cells treated with 33 mM glucose for 5 days. As expected, the mesangial proteins of F-actin and α-SMA were highly induced by the 3-day culture of 33 mM glucose (Figure 2A). In addition, high glucose promoted the formation of F-actin fibers in mesangial cells, which was reduced by 1–20 μM chrysin (Figure 2B). Similarly, the α-SMA level was enhanced in diabetic kidneys, indicating mesangial cell proliferation and expansion (Figure 2C). Unexpectedly, the renal level of F-actin was reduced in diabetic kidneys (Figure 2C). Accordingly, one can assume that glucose-mediated AGE produced mesangial actin polymerization and mesangial proliferation. Increased formation of F-actin fibers may be a cause of renal dysfunction implicated in DN.

### 3.2. Blockade of Focal Adhesion Formation by Chrysin

**Figure 3:**
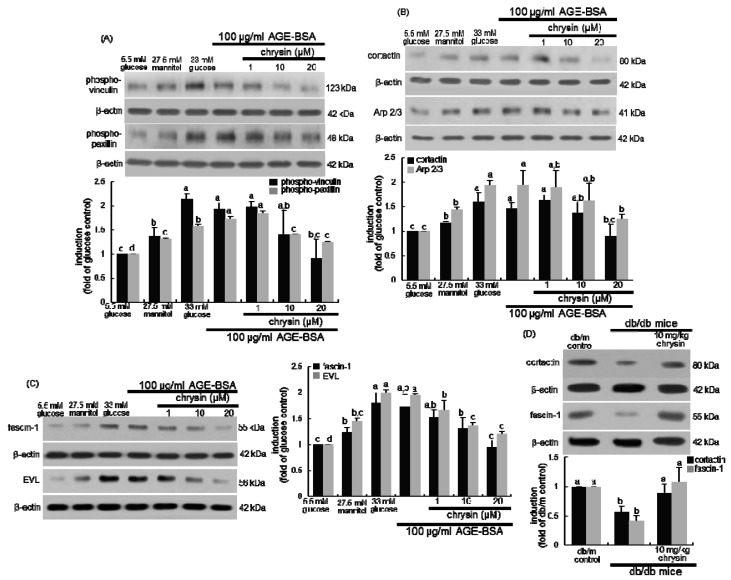
Western blot data showing inhibitory effects of chrysin on AGE-induced cellular induction of phospho-vinculin and phospho-paxillin (**A**), cortactin and Arp2/3 (**B**) and fascin-1 and EVL (**C**) in human renal mesangial cells (HRMC). HRMC were challenged for 3 days with 5.5 mM glucose, 5.5 mM glucose plus 27.5 mM mannitol as osmotic controls, 33 mM glucose or 100 μg/mL AGE-BSA in the absence and presence of 1–20 μM chrysin. *db/db* mice were orally administrated with 10 mg/kg chrysin for 10 weeks (**D**). Cellular induction of phospho-vinculin, phospho-paxillin, cortactin, Arp2/3, fascin-1 and Ena/VASP (EVL) were measured by Western blot analysis with each of their primary antibodies. The renal tissue levels of cortactin and fascin-1 were also measured (**D**). The bar graphs (mean ± SEM, *n* = 3) in the bottom or right panels represent quantitative results obtained from a densitometer. β-Actin protein was used as an internal control. Values not sharing a letter are different at *p* < 0.05.

Dynamics of integrin-based focal adhesions containing F-actin-binding vinculin and paxillin is an unceasing process involving coordination between focal adhesion and the actin cytoskeleton, which is vital for cell migration, polarization, and extracellular matrix remodeling [17,18]. High glucose and AGE-BSA markedly promoted the phosphorylation of vinculin and paxillin in mesangial cells (Figure 3A). The activation of vinculin and paxillin was dose-dependently inhibited by treating 1–20 μM chrysin. Accordingly, chrysin may attenuate the focal adhesion clustering in an active conformation and focal adhesion-actin interaction in the diabetic milieu. 

The F-actin-linked protein cortactin is known to activate the actin polymerization mediated by Arp2/3 complex, a branched actin filament nucleator [35]. The induction of cortactin and ARP2/3 was highly prompted in glucose- or AGE-exposed mesangial cells (Figure 3B). In contrast, such induction dose-dependently declined in 1–20 μM chrysin-exposed mesangial cells. Thus, chrysin may block branched actin network assembly and actin polymerization promoted by glucose.

### 3.3. Inhibition of F-Actin Bundling and Mesangial Migration by Chrysin

Fascin, a filamentous actin-crosslinking/bundling protein, is associated with cell motility and recruited to filopodia [36]. This study examined whether chrysin inhibited the F-actin crosslinking responsible for actin bundling. Both glucose and AGE increased mesangial induction of fascin-1, which was reduced by the presence of ≥10 μM chrysin (Figure 3C). Additionally, chrysin encumbered the induction of EVL involved elongation of actin filaments in glucose- or AGE-treated mesangial cells (Figure 3C). On the other hand, chrysin counteracted the tissue levels of cortactin and fascin-1 highly diminished in diabetic mouse kidneys, along with F-actin (Figure 3D). 

**Figure 4:**
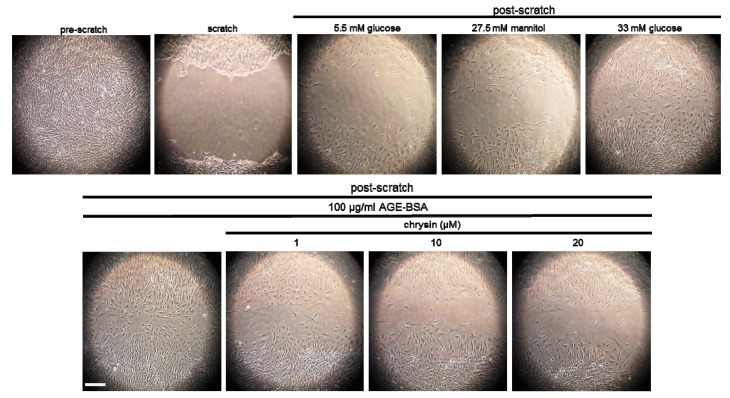
In vitro scratch wound healing assay showing inhibition of migration of glucose- and AGE-exposed human renal mesangial cells (HRMC) by chrysin. HRMC were challenged for 3 days with 5.5 mM glucose, 5.5 mM glucose plus 27.5 mM mannitol as osmotic controls, 33 mM glucose, or 100 μg/mL AGE-BSA in the absence and presence of 1–20 μM chrysin. In vitro scratch wound healing assay was performed (see the Materials and Methods). Scale bar = 200 µm. Each photograph is representative of at least four animals. Magnification: 200-fold.

This study attempted to investigate that chrysin interrupted mesangial cell migration in the diabetic glomerular microenvironment. Mesangial cell motility was enhanced by an exposure to glucose or AGE, evidenced by the in vitro scratch wound healing assay (Figure 4). When the mesangial cells were supplemented with 1–20 μM chrysin, the cell migration was notably attenuated. Accordingly, chrysin may hamper mesangial cell migration through disturbing focal adhesion formation and actin bundling due to glucose-mediated AGE.

### 3.4. Inhibition of Autophagic Responses to AGE by Chrysin

**Figure 5:**
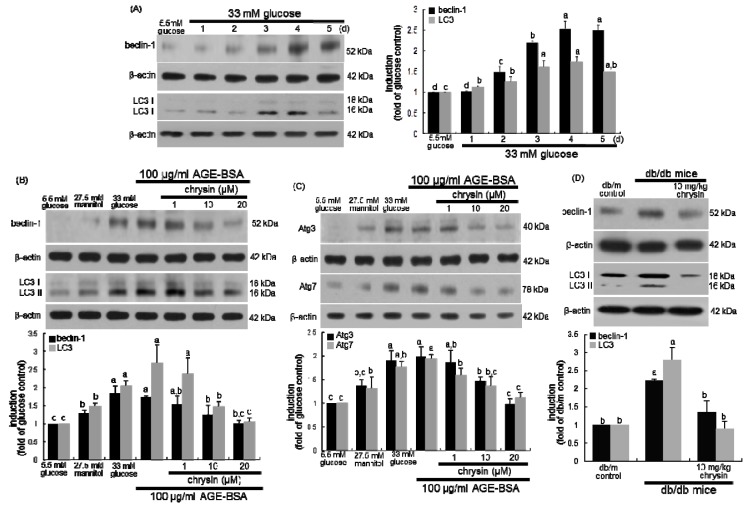
Temporal responses of cellular induction of autophagy-related proteins to high glucose (**A**) and inhibitory effects of chrysin on mesangial induction of autophagy-related proteins by AGE-BSA (**B**,**C**). Human renal mesangial cells were challenged for 3 days with 5.5 mM glucose, 5.5 mM glucose plus 27.5 mM mannitol as osmotic controls, 33 mM glucose or with 100 μg/mL AGE-BSA in the absence and presence of 1–20 μM chrysin. Cellular induction of beclin-1, LC3, Atg3, and Atg7 were measured by Western blot analysis with each of their primary antibodies. *db/db* mice were orally administrated with 10 mg/kg chrysin for 10 weeks. The renal tissue levels of beclin-1 and LC3 were also measured by Western blotting (**D**). The bar graphs (mean ± SEM, *n* = 3) in the right or bottom panels represent quantitative results obtained from a densitometer. β-Actin protein was used as an internal control. Values not sharing a letter are different at *p* < 0.05.

This study investigated that chrysin modulated autophagic activity in mesangial cells placed in the diabetic milieu by evaluating induction of the autophagy markers of beclin-1 and LC3. High glucose enhanced the induction of beclin-1 and LC3 I/II in a temporal manner for 5-day treatment (Figure 5A). Moreover, the induction of beclin-1 and LC3 I/II was observed in mesangial cells exposed to 100 μg/mL AGE-BSA for 4 days (Figure 5B). The increased induction of beclin-1 and LC3 I/II was dose-dependently diminished by chrysin. The induction of the autophagy-related genes of Atg3 and Atg7 was promoted in AGE-exposed mesangial cells, and such induction was reduced in a dose-dependent manner (Figure 5C). Furthermore, renal tissue levels of beclin-1 and LC3 I/II were elevated in diabetic kidneys (Figure 5D). Oral administration of 10 mg/kg chrysin significantly lowered these levels in diabetic mice. Therefore, autophagy activation may contribute to mesangial proliferation and an aberrant actin cytoskeleton, and targeting autophagy genes may be a potential therapeutic strategy. 

### 3.5. Suppression of AGE-Induced mTOR Activation by Chrysin

**Figure 6:**
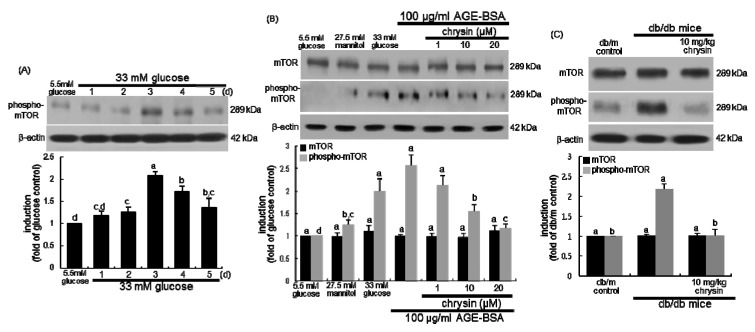
Temporal activation of mTOR to high glucose (**A**), and inhibitory effects of chrysin on its activation in AGE-exposed human renal mesangial cells (HRMC, **B**) and diabetic kidneys (**C**). HRMC were challenged for 3 days with 5.5 mM glucose, 5.5 mM glucose plus 27.5 mM mannitol as osmotic controls, 33 mM glucose or 100 μg/mL AGE-BSA in the absence and presence of 1–20 μM chrysin (**A**,**B**). Diabetic mice were orally administrated with 10 mg/kg chrysin for 10 weeks (**C**). The mTOR phosphorylation in cells and kidney tissues was measured by Western blot analysis with a primary antibody against mTOR or phospho-mTOR. The bar graphs (mean ± SEM, *n* = 3) in the bottom panels represent quantitative results obtained from a densitometer. β-Actin protein was used as an internal control. Values not sharing a letter are different at *p* < 0.05.

An emerging body of evidence has suggested that mTOR protein plays a key role in the development of diabetes [37,38]. This study examined whether chrysin deterred renal mesangial activation of mTOR under diabetic conditions. In 33 mM glucose-exposed mesangial cells for 5 days the mTOR was temporally phosphorylated (Figure 6A). The mTOR phosphorylation increased in mesangial cells subjected to AGE (Figure 6B). In contrast, the increased mTOR activation was attenuated by adding/chrysin to diabetic mesangial cells. When 10 mg/kg chrysin was orally administrated to *db/db* mice, the renal level of phosphorylated mTOR was markedly suppressed (Figure 6C).

### 3.6. Association of Autophagy with Mesangial Motility

**Figure 7:**
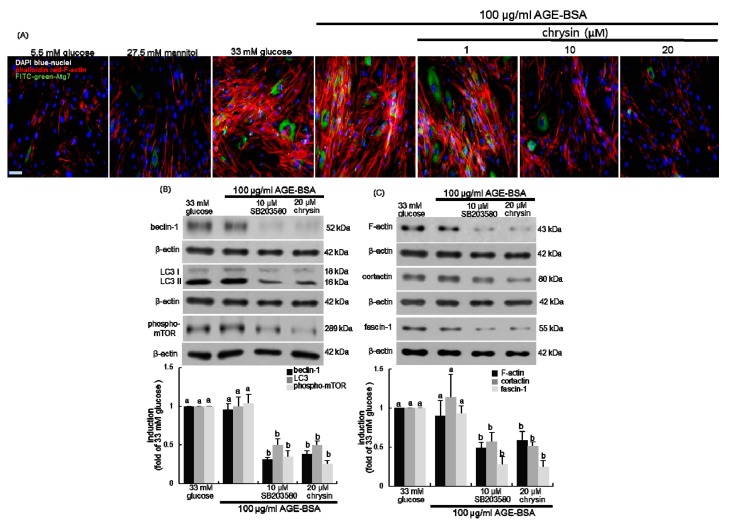
Involvement of autophagy and mTOR in F-actin formation and actin polymerization. Human renal mesangial cells (HRMC) were challenged for 3 days with 5.5 mM glucose, 5.5 mM glucose plus 27.5 mM mannitol as osmotic controls, 33 mM glucose or 100 μg/mL AGE-BSA in the absence and presence of 1–20 μM chrysin or 10 μM SB203580. The F-actin formation and Atg7 induction in glucose- or AGE-exposed HRMC were visualized with rhodamine phalloidine-red staining and FITC-green immunostaining, and nuclear counter-staining was done with the blue stain 4’,6-diamidino-2-phenylindole (**A**). Each photograph is representative of four different experiments and images were taken with a fluorescence microscope. 200-fold. Scale bar = 100 µm. Cellular induction of beclin-1, LC3, phospho-mTOR, F-actin, cortactin, and fascin-1 were measured by Western blot analysis with each of their primary antibodies (**B**,**C**). The bar graphs (mean ± SEM, *n* = 3) in the bottom panels represent quantitative results obtained from a densitometer. β-Actin protein was used as an internal control. Values not sharing a letter are different at *p* < 0.05.

A recent paper has reviewed the cytoskeleton-autophagy connection [39]. The current study elucidated that Atg7 contributed to actin assembly under pathological diabetic conditions. The coexisting induction of both F-actin and Atg7 was highly augmented in 33 mM glucose- or 100 μg/mL AGE-BSA-treated mesangial cells, indicating that green FITC-Atg7 co-localized with red phalloidin-F-actin in mesangial cells (Figure 7A). However, the presence of chrysin attenuated the induction of Atg7 localized to the actin cytoskeleton in mesangial cells (Figure 7A). 

Since p38 MAPK regulates distinct phases of autophagy [40,41], this study employed the p38 MAPK inhibitor for the inhibition of mesangial autophagy. As expected, the beclin-1 induction and LC3 I/II activation by AGE was near-completely abated in mesangial cells by 10 μM SB203580, a p38 MAPK inhibitor (Figure 7B). In addition, the mesangial mTOR activation by AGE was abrogated by the inhibition of p38 MAPK (Figure 7B). Accordingly, p38 MAPK may elicit autophagy via beclin-1 induction and LC3 I/II activation. This study further investigated that autophagy and mTOR activation were involved in mesangial protrusion and migration under diabetic conditions. The induction of mesangial F-actin, cortactin, and fascin-1 was dampened by the inhibition of p38 MAPK, as with the presence of 20 μM chrysin (Figure 7C). Therefore, chrysin may inhibit mesangial actin assembly and cell migration by interfering with autophagy via the blockade of beclin-1 and LC3 I/II. 

## 4. Discussion

Ten major findings were observed in this study. (1) The enhanced mesangial F-actin induction and bundle formation by 33 mM glucose or 100 μg/mL AGE-BSA were attenuated by the presence of ≥10 μM chrysin in mesangial cells. (2) The mesangial induction of α-SMA by glucose and the tissue level of α-SMA in diabetic kidneys were reduced by chrysin, indicating its blockade of mesangial proliferation. (3) Treating chrysin inhibited the activation of vinculin and paxillin and the induction of cortactin, ARP2/3, fascin-1, and EVL in glucose- or AGE-exposed mesangial cells. (4) Oral treatment of 10 mg/kg chrysin diminished renal tissue levels of cortactin and fascin-1 elevated in diabetic mouse kidneys. (5) Mesangial cell motility was enhanced in glucose- or AGE-exposed mesangial cells, which was notably attenuated by adding chrysin. (6) Chrysin dampened the induction of autophagy-related genes of beclin-1, LC3 I/II, Atg3, and Atg7 in mesangial cells exposed to AGE. (7) Oral administration of chrysin lessened the tissue levels of beclin-1 and LC3 I/II enhanced in diabetic kidneys. (8) Chrysin reduced the mTOR activation in AGE-exposed mesangial cells and diabetic kidneys. (9) The presence of chrysin attenuated the induction of Atg7 localized to the F-actin cytoskeleton in mesangial cells. (10) The induction of mesangial F-actin, cortactin, and fascin-1 by AGE was near-completely abolished by the inhibition of autophagy. Therefore, chrysin may hamper diabetes-associated mesangial cell protrusion and migration through disturbing actin dynamics by interfering with autophagy via the blockade of beclin-1 and LC3 I/II. 

The irregular mesangial structures in kidney glomeruli influence the blood capillary flow through manipulating expansible forces created by pressure gradients in glomerular vessels [4]. A growing body of evidence shows that the aberrant proliferation and migratory polarity of mesangial cells plays a role in the pathogenesis of glomerulonephritis due to glomerular injury [4,7,8]. However, the underlying molecular changes for mesangial aberrant proliferation and motility during glomerular injury remain elusive. The cell migratory process is controlled by well-coordinated actin dynamics and focal adhesion turnover at the peripheral ruffles in migrating cells [6]. Numerous studies have shown that glomerular mesangial cells migrate in response to pathophysiological stimuli such as PDGF, angiotensin II, oxidants and inflammatory cytokines [5,6]. Accordingly, alterations in induction and localization of cytoskeletal regulators trigger mesangial phenotypic alterations in glomerulonephritis [6]. However, identifying the key molecular components steering motility and actin functions in mesangial cells remains challenging. This study investigated the mesangial dynamics of F-actin cytoskeleton and integrin-based focal adhesions in glomerular injury due to glucose and AGE implicated in the pathogenesis of diabetic complications. It was found that glucose and AGE prompted aberrant F-actin polymerization, bundling, and motility in mesangial cells. AGE induces alterations in cell morphology and function by affecting the cytoskeletal structure through activation of the receptor for AGE (RAGE) [14,15].

Several studies have demonstrated that actin-targeting natural plant compounds influence actin dynamics, prompting either polymerization or depolymerization in signaling pathways through diverse mechanisms [42,43]. A recent study shows that salvianolic acid A inhibits renal cytoskeletal dysfunction through inhibiting the AGE–RAGE signaling, thus effectively ameliorating early-stage DN [44]. The polyphenol honokiol counteracts with cisplatin-induced cytoskeletal structure disruption in renal epithelial cells and preserves epithelial cell polarity and morphology [45]. Our previous study found that chrysin, a naturally-occurring flavonoid, inhibited AGE-induced kidney fibrosis in renal mesangial cells and diabetic kidneys through inhibition of AGE–RAGE activation, contributing to blockade of glucose-mediated AGE-associated glomerulosclerosis and fibrosis [34]. The current study examined whether chrysin inhibited glucose- and AGE-stimulated actin cytoskeleton rearrangement and focal adhesion dysfunction in renal mesangial cells. Herein, chrysin diminished the F-actin fiber formation highly enhanced by glucose and AGE. Chrysin attenuated the activation of F-actin-binding focal adhesion proteins of vinculin and paxillin in diabetic mesangial cells. This study also found that this compound encumbered the mesangial cell induction of cortactin, Arp2/3 complex, EVL, and fascin-1, all involved in actin polymerization and actin-crosslinking/bundling. The EVL proteins enhance in the cellular processes including axon guidance and cell migration as modulators of the actin cytoskeleton and cell migration [46]. Unexpectedly, the renal tissue levels of F-actin, cortactin, and fascin-1 were reduced in diabetic mouse kidney, which was counteracted by chrysin. The kidney is composed of several different types of cells such as podocytes and glomerular vascular cells, and the mesangial cell population in the whole kidney is very small. Accordingly, the overall renal tissue levels of F-actin, cortactin, and fascin-1 might be lowered in diabetic kidneys comprised of diverse types of glomerular cells. Unlike mesangial cells, the induction of F-actin and cortactin was reduced in high glucose-exposed podocytes. Nevertheless, these findings shed light on a therapeutic role for chrysin in modulating aberrant actin remodeling and abnormal coordination between F-actin and focal adhesion in mesangial cells during glucose or AGE-induced glomerular injury. 

Autophagy serves as a vital mechanism to maintain kidney homeostasis, and its impairment is implicated in the pathogenesis of DN [47]. One investigation shows that autophagy is involved in tumor cell motility and focal adhesion disassembly through targeted degradation of paxillin interacting with processed LC3 [48]. The autophagy inhibition reduces tumor cell migration and invasion and attenuates metastasis [48]. The present study demonstrated that glucose and AGE enhanced mesangial cell autophagy through inducing the beclin-1-LC3 I/II-Atg pathway, and that chrysin encumbered such diabetic induction of autophagy. This study further attempted to reveal that autophagy contributed to F-actin polymerization/bundling and focal adhesion dysfunction in the diabetic milieu. The presence of chrysin in mesangial cells reduced the induction of Atg7 localized to the F-actin cytoskeleton. Consistently, one study shows that a mouse Atg7 knockout inhibiting autophagosome formation displays severe defects in actin assembly owing to reduced expression of proteins involved in controlling actin dynamics [49]. Additionally, the inhibition of autophagy deterred the induction of mesangial F-actin, cortactin, and fascin-1 by AGE, indicating that actin polymerization and bundling entailed autophagic activity. On the other hand, chrysin suppressed the mTOR activation in AGE-exposed mesangial cells and diabetic kidneys. Similarly, one study shows that paeoniflorin attenuates AGE-induced mesangial cell damage through inhibiting the RAGE/mTOR/autophagy pathway [50]. Moreover, the blockade of mTOR activation highly attenuated the mesangial induction of F-actin, cortactin, and fascin-1. Growing evidence has suggested the interconnections of mTOR to autophagy in cellular signaling pathways in kidneys [51]. Unfortunately, this study did not examine reciprocal interconnection of mTOR to autophagy in mesangial proliferation and motility due to AGE. Inhibition of mTOR attenuates the actin crosslinking protein filamin A-dependent focal adhesion formation and cell migration [52]. 

## 5. Conclusions

This study elucidated that chrysin influenced glucose- or AGE-stimulated actin polymerization, focal adhesion disassembly and cell migration contributing to phenotypic alterations in mesangial cells. These diabetic stimuli induced mesangial cell proliferation, aberrant actin cytoskeleton, and focal adhesion formation. However, chrysin ameliorated dysregulation of mesangial actin assembly and bundling through blocking the induction of F-actin, cortactin, EVL, and fascin-1. In addition, this flavonoid hampered diabetic AGE-enhanced formation of focal adhesions involving paxillin and vinculin. Furthermore, the autophagy and mTOR pathways were involved in actin polymerization, focal adhesion disassembly and cell migration in AGE-stimulated mesangial cells. Therefore, chrysin may counteract diabetes-associated mesangial cell protrusion and migration through disturbing actin polymerization/bundling and focal adhesion formation by interfering with autophagy/mTOR activation. Since the mesangial cell population is very small in whole kidneys, the dissociation between the in vitro and in vivo results may take place. Accordingly, the immunohistochemical sequential double staining is needed to examine the mesangial level of F-actin, cortactin or fascin-1 in kidneys, along with mesangial α-SMA level. Western blotting with primary culture mesangial cells or glomeruli can be considered by using sieving methods at least cortex part of kidneys.
